# The association between adiponectin gene rs182052 polymorphism and cancer risk: a meta-analysis

**DOI:** 10.1042/BSR20192410

**Published:** 2020-06-26

**Authors:** Li-Fang Wu, Gui-Ping Xu, Qing Zhao, Ding Wang, Li-Jing Zhou, Bin Sun, Wei-Xian Chen

**Affiliations:** 1 Department of Laboratory Medicine, The Second Affiliated Hospital of Chongqing Medical University, Chongqing, China; 2 Transfusion Department, The Second Affiliated Hospital of Chongqing Medical University, Chongqing, China

**Keywords:** adiponectin, cancer, meta-analysis, polymorphism, rs182052

## Abstract

**Background:** The evidence for an association between the *adiponectin* gene *(ADIPOQ)* polymorphism rs182052 and cancer risk is inconsistent. We performed a meta-analysis to obtain more precise conclusions.

**Methods:** The PubMed, Embase, and Web of Science databases were searched until July 11, 2019. And seven epidemiology studies were retrieved, including 4,929 cases and 5,625 controls. Odds ratios (ORs) and the corresponding 95% confidence intervals (CIs) were calculated to evaluate the strength of the association.

**Results:** The meta-analysis demonstrated that rs182052 significantly increased the risk of cancer under the allele, homozygote, dominant, and recessive models, based on an overall analysis (A vs. G: OR, 1.09, 95% CI, 1.03–1.15, *P*=0.003; AA vs. GG: OR, 1.20, 95% CI, 1.07–1.34, *P*=0.002; AA+GA vs. GG: OR, 1.12, 95% CI, 1.03–1.22, *P*=0.010; AA vs. GA+GG: OR, 1.12, 95% CI, 1.01–1.23, *P*=0.025). In the stratified analysis by ethnicity, rs182052 significantly increased the cancer risk in both Asian and Caucasian populations under one or several genetic models. In the stratified analysis by cancer type, rs182052 significantly increased the risk of renal cell carcinoma (RCC) under the five models.

**Conclusions:** Meta-analysis based on present studies suggests that rs182052 can increase the cancer risk.

## Introduction

Obesity has become a global public health issue, and the number of overweight and obese individuals has been increasing worldwide in recent years [1,2]. Obesity has been related to a variety of diseases, including metabolic disease, cardiovascular diseases, and cancer [3,4]. The mechanism linking obesity and cancer is not completely understood, adiponectin has been reported as one of the molecular mediators [5,6].

Adiponectin is an adipokine produced mainly by white adipose tissue, and circulating adiponectin levels are reduced in overweight or obese people [7]. A low level of circulating adiponectin is also significantly associated with an increased risk of various types of cancer [8–11]. The biological functions of adiponectin include anti-inflammatory, anti-proliferative, and pro-apoptotic effects [12,13]. Adiponectin exerts its anti-cancer effects through multiple pathways, the most important being the activation of adenosine monophosphate-activated protein kinase (AMPK) [14,15]. The decreasing of adiponectin also reduced the synthesizing of insulin-like growth factor binding protein 1 (IGFBP1) and insulin-like growth factor binding protein 2 (IGFBP2) in the liver, thereby increasing bioavailability of insulin-like growth factor 1 (IGF1), and contributing to cancer development [16].

The rs182052 polymorphism is positioned at the *adiponectin* gene *(ADIPOQ)* promoter region, and is associated with adiponectin levels as well as risks of a variety of cancers [16–22], such as prostate, colorectal, breast, and kidney cancer. However, the association between rs182052 and cancer remains controversial. For example, Dhillon et al. reported a significant association between rs182052 and prostate cancer risk [18], whereas Moore et al. concluded that rs182052 was not related to prostate cancer risk [17]. The aim of the present study was to summarize the existing epidemiological studies and obtain precise conclusions by performing the meta-analysis.

## Methods

### Search strategy

The Pubmed, Embase, and Web of Science databases were searched for the possible studies that investigated the relationship between rs182052 and cancer risk until July 11, 2019.

The key search words were: “*adiponectin* or *ADIPOQ*,” “mutation or variant or polymorphism or SNP,” and “cancer or carcinoma or tumor.” We also checked the references of selected studies for possible related studies.

### Inclusion and exclusion criteria

The inclusion criteria were:

1. The studies should be about the relationship between rs182052 and cancer;
2. The studies should be case–control or cohort designed;
3. The studies should contain sufficient genotype data for meta-analysis; and
4. The studies were published in English.

We excluded reviews or meta-analyses and any studies not containing sufficient genotype data.

### Data extraction

The following data were extracted from the selected studies by two authors separately: name of first author, year published, country or region, ethnicities of the population, genotype method, control source, and genotype frequency.

### Quality score

The quality of the included studies was scored according to the following factors [23]: source of case, source of control, number of subjects, and Hardy–Weinberg equilibrium (HWE) (Supplementary Table S1).

### Statistical analysis

We evaluated the strength of the associations by OR and 95% Cl under five genetic models: the allele, homozygote, heterozygote, dominant, and recessive models. *P* values <0.05 were considered statistically significant. Heterogeneity was assessed by the Chi-squared test and Higgins’s (*I*^2^) test [24]. If *I*^2^ < 50% or the *P*-value of heterogeneity was > 0.10, the fixed-effects model was used [25]; otherwise, random effects was used [26]. We conducted stratified analysis based on the following factors: ethnicity, cancer type, and quality score. For sensitivity analysis, we took a strategy of removing one study each time [27]. The potential publication bias was evaluated using the Egger’s or Begg’s test [28,29]. All statistical analyses were performed using STATA software (Version 12.0, Stata Corporation, College Station, TX).

### Trial sequential analysis and false-positive report probability analysis

Trial sequential analysis (TSA) and false-positive report probability (FPRP) analysis were performed as reported previously [30–32]. Briefly, TSA was performed using the TSA v0.9.5.10 beta software. In the present study, we set the type-I error to 5%, the statistical test power to 80%, and the relative risk reduction to 20%. FPRP values were calculated using the approach developed by Wacholder et al. [33]. We set the FPRP threshold at 0.2 and the prior probability at 0.1. Only FPRP values less than 0.2 were considered to indicate a noteworthy association.

## Results

### Characteristics of the studies

**Figure 1:**
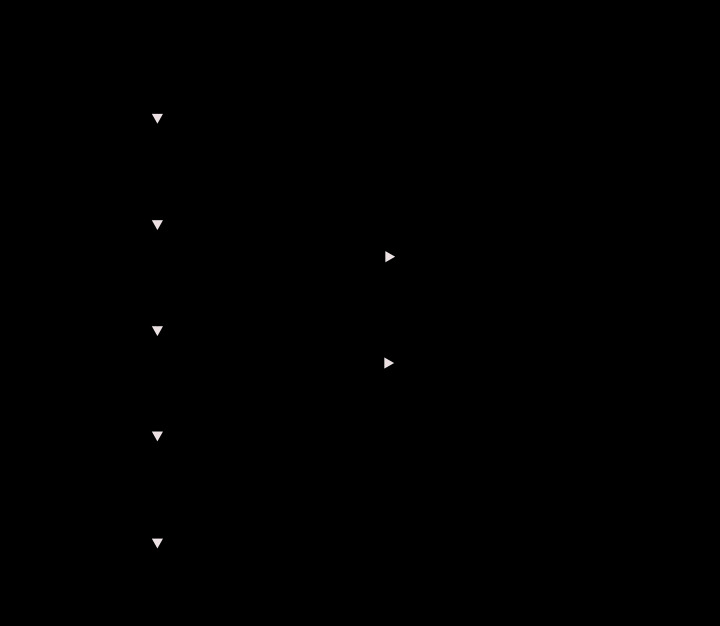
The flow diagram of included/excluded studies

The process of article screening is shown in Figure 1. We obtained 298 articles by searching the databases. By reading titles and abstracts, we deleted 268 records and 30 articles for full-text reading. We then screened those 30 articles according to our inclusion and exclusion criteria, and ultimately selected seven articles for the meta-analysis. These seven included articles were published between 2009 and 2018; four were conducted on the Asian population, two on the Caucasian population, and one on a mixed population. The characteristics of the studies are listed in Table 1.The allele and genotype frequencies are shown in Table 2.

**Table 1:** Characteristics of the studies included in the meta-analysis

First author	Year	Country/Region	Ethnicity	Cancer type	Genotyping method	Control source
Moore [17]	2009	Southwestern Finland	Mix	Prostate cancer	Taqman	PB
Al Khaldi [16]	2011	Kuwait	Caucasian	Breast, prostate and colon cancer	Taqman	Blood donors
Dhillon [18]	2011	U.S.A.	Caucasian	Prostate cancer	MALDI-TOF MS	HB
Gu [19]	2014	China	Asian	Prostate cancer	Taqman	HB
Zhang [20]	2015	China	Asian	RCC	Taqman	HB
Park [21]	2015	Korea	Asian	Colorectal cancer	Human SNP array 5.0	PB
Hsueh [22]	2018	Taiwan	Asian	RCC	PCR-RFLP	HB

Abbreviations: HB, hospital-based; MALDI-TOF MS, matrix-assisted laser desorption/ionization time-of-flight mass spectrometry; PB, population-based; PCR-RFLP, PCR restriction fragment length polymorphism; RCC, renal cell carcinoma.

**Table 2:** *ADIPOQ* rs182052 polymorphism genotype distribution and allele frequency in cases and controls

	Genotype (N)								Allele frequency (N)				HWE	Score
	Case				Control				Case		Control			
	Total	GG	GA	AA	Total	GG	GA	AA	G	A	G	A		
Moore [17]	943	205	472	266	854	202	400	252	882	1004	804	904	0.079	15
Al Khaldi [16]	132	23	101	8	68	12	52	4	147	117	76	60	<0.001	8
Dhillon [18]	1219	545	527	147	1196	564	524	108	1617	821	1652	740	0.535	12
Gu [19]	917	264	448	205	1036	279	514	243	976	858	1072	1000	0.834	12
Zhang [20]	1004	249	485	270	1108	315	544	249	983	1025	1174	1042	0.628	12
Park [21]	325	74	165	86	974	255	485	234	313	337	995	953	0.909	15
Hsueh [22]	389	113	194	82	389	147	178	64	420	358	472	306	0.417	11

Abbreviation: HWE, Hardy–Weinberg equilibrium.

### Meta-analysis

**Figure 2:**
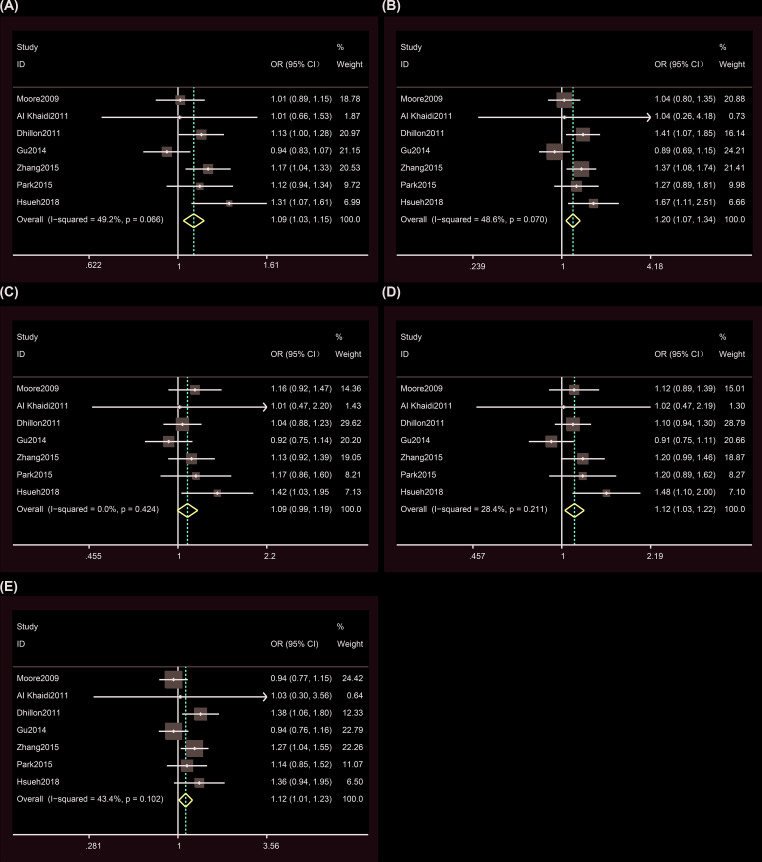
Meta-analysis of the association between rs182052 and risk of cancer (**A**) Allele model; (**B**) homozygous model; (**C**) heterozygous model; (**D**) dominant model; (**E**) recessive model. The squares and horizontal lines correspond to the study specific OR and 95% CI. The area of the squares reflects the weight. The diamond represents the summary OR and 95% CI. The fixed-effects model was used.

In total, the meta-analysis consisted of 4929 cases and 5625 controls. The synthesis results demonstrated that rs182052 increased the risk of cancer under the allele, homozygote, dominant, and recessive models in the overall analysis (Table 3 and Figure 2, A vs. G: OR, 1.09, 95% CI, 1.03–1.15, *P*=0.003; AA vs. GG: OR, 1.20, 95% CI, 1.07–1.34, *P*=0.002; AA+GA vs. GG: OR, 1.12, 95% CI, 1.03–1.22, *P*=0.010, AA vs. GA+GG: OR, 1.12, 95% CI, 1.01–1.23, *P*=0.025).

**Table 3:** Meta-analysis of the association between rs182052 polymorphism and cancer susceptibility

Subgroup	No.	A vs. G			AA vs. GG			GA vs. GG			AA + GA vs. GG			AA vs. GA + GG		
		OR (95%Cl)	*P*_OR_	*I*^2^	OR (95%Cl)	*P*_OR_	*I*^2^	OR (95%Cl)	*P*_OR_	*I*^2^	OR (95%Cl)	*P*_OR_	*I*^2^	OR (95%Cl)	*P*_OR_	*I*^2^
Overall	7	**1.09 (1.03–1.15)**	**0.003**	49.2%	**1.20 (1.07–1.34)**	**0.002**	48.6%	1.09 (0.99–1.19)	0.069	0.0%	**1.12 (1.03–1.22)**	**0.010**	28.4%	**1.12 (1.01–1.23)**	**0.025**	43.4%
Asian	4	1.12 (0.97–1.29)*	0.112	70.0%	1.24 (0.95–1.62)*	0.113	67.3%	1.10 (0.97–1.24)	0.144	44.2%	1.16 (0.95–1.42)^*^	0.141	63.7%	**1.14 (1.01–1.28)**	**0.041**	42.4%
Caucasian	2	**1.12 (1.00–1.26)**	**0.049**	0.0%	**1.39 (1.06–1.82)**	**0.016**	0.0%	1.04 (0.88–1.23)	0.645	0.0%	1.10 (0.94–1.29)	0.234	0.0%	**1.36 (1.06–1.76)**	**0.018**	0.0%
Prostate cancer	3	1.03 (0.92–1.15)^*^	0.617	54.5%	1.09 (0.84–1.41)^*^	0.525	66.2%	1.03 (0.92–1.16)	0.609	5.3%	1.05 (0.94–1.17)	0.428	23.2%	1.05 (0.84–1.33)^_^	0.654	68.4%
RCC	2	**1.21 (1.09–1.34)**	**<0.001**	0.0%	**1.44 (1.17–1.77)**	**0.001**	0.0%	**1.21 (1.02–1.44)**	**0.033**	28.3%	**1.28 (1.09–1.51)**	**0.003**	23.7%	**1.29 (1.08–1.53)**	**0.004**	0.0%
Quality score≥10	6	1.10 (1.01–1.20)^*^	**0.037**	57.2%	1.22 (1.02–1.46)^*^	**0.029**	57.0%	1.09 (0.99–1.19)	0.068	16.1%	**1.12 (1.03–1.22)**	**0.010**	39.9%	1.13 (0.98–1.31)^_^	0.089	52.7%

Abbreviations: 95% CI, 95% confidence interval; OR, odds ratio; *P*_OR_, pool *P* value; RCC, renal cell carcinoma; *indicates that the OR, 95% Cl, and corresponding *P*_OR_ were calculated based on the random-effects model; otherwise, the fixed-effects model was used. Bold values are statistically significant (*P*_OR_ < 0.05).

In the stratified analysis based on ethnicity, rs182052 increased the risk of cancer in the Asian population under the recessive model (Table 3, AA vs. GA+GG: OR, 1.14, 95% CI, 1.01–1.28, *P*=0.041), and increased the risk of cancer in the Caucasian population under the allele, homozygote, and recessive models (Table 3, A vs. G: OR, 1.12, 95% CI, 1.00–1.26, *P*=0.049; AA vs GG: OR, 1.39, 95% CI, 1.06–1.82, *P*=0.016; AA vs. GA+GG: OR, 1.36, 95% CI, 1.06–1.76, *P*=0.018). In the stratified analysis based on cancer type, rs182052 increased the risk of renal cell carcinoma (RCC) under all five genetic models (Table 3, A vs. G: OR, 1.21, 95% CI, 1.09–1.34, *P*<0.001; AA vs. GG: OR, 1.44, 95% CI, 1.17–1.77, *P*=0.001; GA vs. GG: OR, 1.21, 95% CI, 1.02–1.44, *P*=0.033; AA+GA vs. GG: OR, 1.28, 95% CI, 1.09–1.51, *P*=0.003, AA vs. GA+GG: OR, 1.29, 95% CI, 1.08–1.53, *P*=0.004). We found no evidence to support a relationship between rs182052 and prostate cancer risk. The results of stratified analysis based on the quality score showed that rs182052 increased the risk of cancer under the allele, homozygote, and dominant models when the score was less than 10 (Table 3, A vs. G: OR, 1.10, 95% CI, 1.01–1.20, *P*=0.037; AA vs. GG: OR, 1.22, 95% CI, 1.02–1.46, *P*=0.029; AA+GA vs. GG: OR, 1.12, 95% CI, 1.03–1.22, *P*=0.010).

### Sensitivity analysis

**Figure 3:**
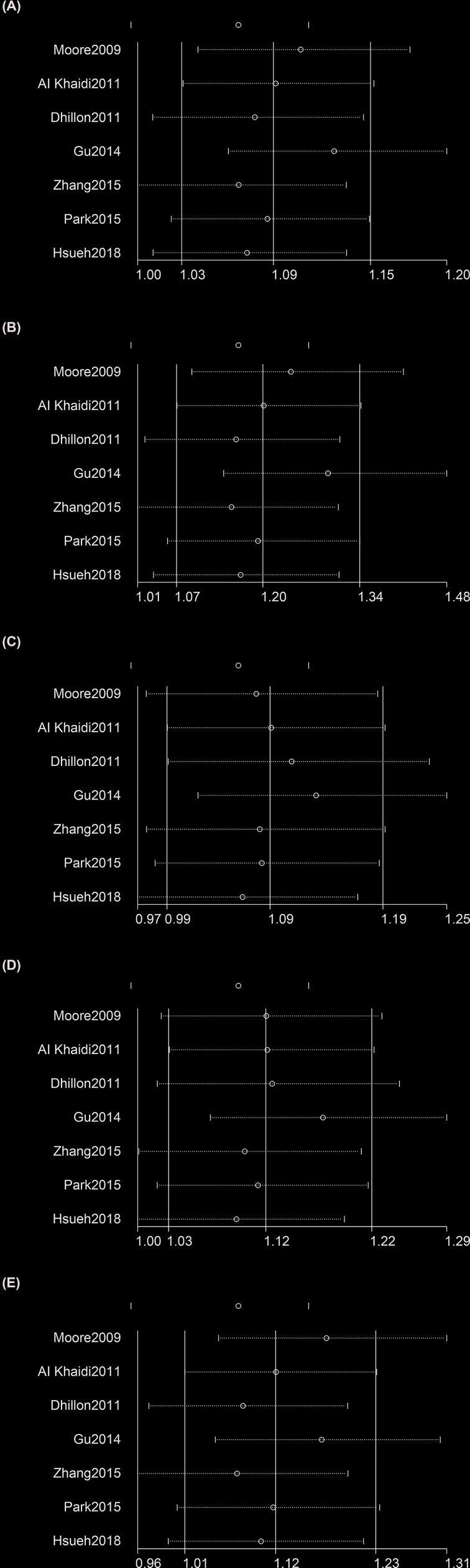
Sensitivity analyses between rs182052 polymorphism and risk of cancer (**A**) Allele model; (**B**) homozygous model; (**C**) heterozygous model; (**D**) dominant model; (**E**) recessive model**.** The fixed-effects model was used.

We performed a sensitivity analysis using the *metainf* command in the Stata software. The strategy of this analysis using this command is to remove one study each time. The results of removal of the study by Gu et al. under the heterozygote model showed that rs182052 increase the cancer risk (Figure 3 and Supplementary Table S2). This result validates the important role of rs182052 in cancer risk. The results of removal of the study by Hsueh et al. under the dominant model and the removal of the study by Dhillon, Zhang, or Hsueh et al. under the recessive model showed rs182052 could not increase the cancer risk (Figure 3 and Supplementary Table S2). These differences demonstrate that our results were not stable under the two models and that they need further validation.

### Publication bias

Publication bias was checked using Begg’s test and Egger’s test. We did not detect publication bias in the meta-analysis (Table 4).

**Table 4:** Publication bias analysis

Genetic model	Egger’s test			Begg’s test
	*T*	95% Cl	*P*	*P*
A vs. G	0.34	−4.206–5.508	0.744	1.000
AA vs. GG	0.40	−3.577–4.906	0.704	0.548
GA vs. GG	0.82	−2.013–3.912	0.448	0.368
AA+GA vs. GG	0.55	−2.822–4.356	0.606	0.764
AA vs. GA+GG	0.49	−3.144–4.608	0.648	1.000

### TSA and FPRP analyses

**Figure 4:**
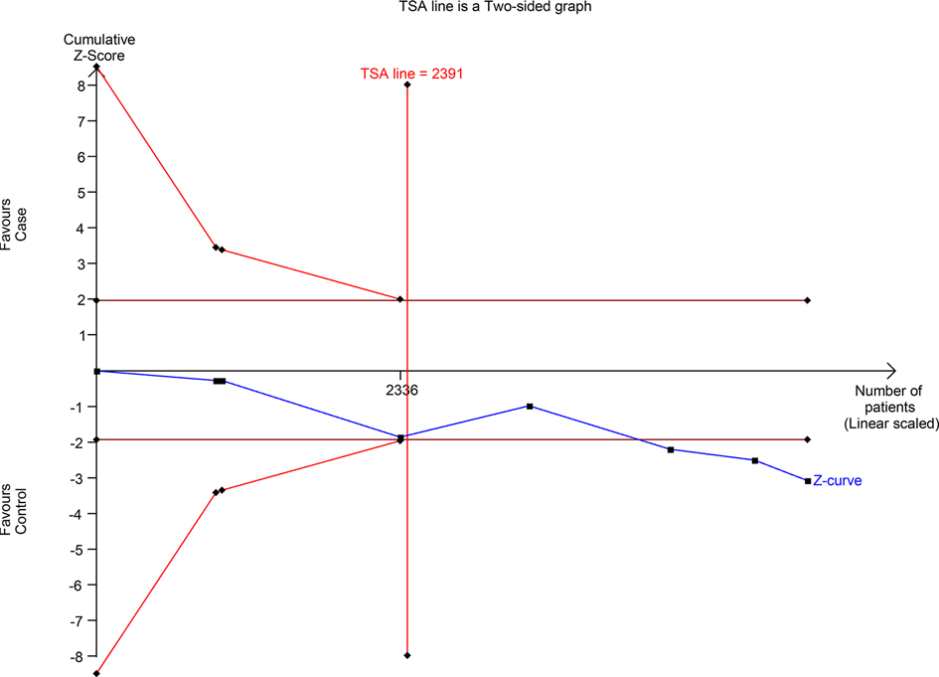
TSA of the association between rs182052 polymorphism and cancer risk under the homozygous model

We performed the TSA in the overall analysis under the homozygote model (Figure 4). The cumulative *Z*-curve crossed the conventional boundary for significance, which was consistent with the meta-analysis results. Although the cumulative *Z*-curve did not cross any trial sequential monitoring boundary, the cumulative *Z*-curve reached the required information size, indicating that the accumulated sample size was sufficient and the result was credible.

We conducted FPRP analysis for all the significant associations in the overall analysis (Supplementary Table S3). When the prior probability was 0.1, these FPRP values were all <0.2, indicating that the associations were noteworthy.

## Discussion

In recent years, obesity has become one of the major public health challenges in the world [34,35]. A study conducted in 2014,based on 19.2 million adults in 186 countries, demonstrated that the percentage of obese people in the world increased from 3.2% in 1975 to 10.8% in 2014 for men, and from 6.4% to 14.9% for women [36]. Obesity is related to many health problems, such as high blood pressure, kidney disease, and cardiovascular disease [37,38]. Many prospective studies have also reported that overweight and obesity are related to the development and prognosis of various types of cancer [39,40]; however, its molecular mechanism is still unclear. Adiponectin is recognized as one of the key mediating factor involved in the cancer link to obesity [41]. Circulating adiponectin levels are inversely associated with cancer risk [42].

Several previous meta-analyses have focused on the associations between *ADIPOQ* polymorphisms and cancer risk [43–45]. For example, Zhou et al. reported an association between the *ADIPOQ* rs2241766 G allele and a significantly reduced cancer risk [43]. Similarly, Li et al. suggested that *ADIPOQ* rs1501299 was a protective polymorphism from cancer [44]. Ye et al. demonstrated that both rs2241766 and rs1501299 could reduce cancer risk in the Chinese and Ashkenazi Jewish populations [45].

The rs182052 polymorphism is located in intron 1 of the *ADIPOQ* promoter region, with the minimum gene frequencies (MAFs) greater than 5% in most populations of the 1000 Genome Project (Supplementary Table S4). It has been reported that the rs182052 polymorphism A allele was associated with lower levels of adiponectin [46]. One study reported that rs182052 was associated with body mass index, waist circumference, weight–height ratio, and subcutaneous fat levels in the Hispanic population [47]. Several studies have reported that rs182052 is related to the risk of cancer, but the results are inconsistent. We conducted this meta-analysis to obtain a more definitive conclusion.

The results of our meta-analysis suggested that rs182052 significantly increased the risk of cancer in the overall analysis. The results of the stratified analysis by ethnicity showed that rs182052 significantly increased the cancer risk in both Asian and Caucasian populations. The results of the stratified analysis by cancer type showed that rs182052 significantly increased the risk of RCC. The presence of rs182052 was not related to prostate cancer risk according to the synthesis results of our meta-analysis. Our research provides clues for detecting the molecular mechanism of the function of *ADIPOQ* in cancer. Our findings may also be helpful in developing new molecular monitor indicators of cancer risk and in providing a new theoretical basis for the prevention of cancer for the risk genotype population.

Some limitations of the meta-analysis need to be considered and further explored. First, only five types of cancer were included in our meta-analysis, so, whether the conclusions drawn from these studies can represent the overall cancer risk need to be further explored; second, due to the limited number of subject, we did not consider other factors, such as BMI, and we did not conduct stratified analysis based on these factors. Third, Many SNPs have been reported to be related to cancer risk because the polymorphisms could affect the mRNA expression of the gene [48]. The mechanism by which rs182052 causes an increased risk of cancer is unclear, but the possibility that rs182052 affects cancer risk by affecting *adiponectin* mRNA expression requires further research.

In conclusion, the synthesis results based on current existing studies suggest that rs182052 can increase the risk of cancer.

## Supplementary Material

Supplementary Tables S1-S4

## Abbreviations

ADIPOQ: adiponectin gene
AMPK: adenosine monophosphate-activated protein kinase
CI: confidence interval
FPRP: false-positive report probability
HB: hospital-based
HWE: Hardy–Weinberg equilibrium
IGF1: insulin-like growth factor
IGFBP1: insulin-like growth factor binding protein 1
IGFBP2: insulin-like growth factor binding protein 2
MAF: minor allele frequency
MALDI-TOF MS: matrix-assisted laser desorption/ionization time-of-flight mass spectrometry
OR: odds ratio
PB: population-based
PCR-RFLP: PCR restriction fragment length polymorphism
RCC: renal cell carcinoma
TSA: trial sequential analysis
