# Supplementary material for: The association between adiponectin gene rs182052 polymorphism and cancer risk: a meta-analysis
Source: Biosci Rep. 2020 Jun 26;40(6):BSR20192410. doi: 10.1042/BSR20192410 (PMC7322108; doi:10.1042/BSR20192410)
Supplement: Supplementary Tables S1-S4 [file BSR-2019-2410_supp.pdf]

Scale for quality assessment criterion (23)

	Criterion	Score
**A**	**Source of cases**	
	Selected from population or cancer registry	3
	Selected from hospital	2
	Selected from pathology archives, but without description	1
	Not described	0
**B**	**Source of controls**	
	Population-based	3
	Blood donors or volunteers	2
	Hospital-based (cancer-free patients)	1
	Not described	0
**C**	**Specimens used for determining genotypes**	
	White blood cells or normal tissues	3
	Not mentioned	2
	Tumor tissues or exfoliated cells of tissue	0
**D**	**Hardy–Weinberg equilibrium in controls**	
	Hardy–Weinberg equilibrium	3
	Hardy–Weinberg disequilibrium	0
**E**	**Total sample size**	
	≥1,000	3
	≥500 and <1,000	2
	≥200 and <500	1
	<200	0

Table S1: Quality score assessment

	**A**	**B**	**C**	**D**	**E**	**Score**
Moore 2009	3	3	3	3	3	15
Al Khaldi 2011	2	2	3	0	1	8
Dhillon2011	2	1	3	3	3	12
Gu 2014	2	1	3	3	3	12
Zhang 2015	2	1	3	3	3	12
Park2015	3	3	3	3	3	15
Hsueh 2018	2	1	3	3	2	11

A-E represents the corresponding criterion in the table of scale for quality assessment criterion.

Table S2:Sensitivity analyses for rs182052polymorphism and cancer susceptibility

Comparison	Study omitted	Estimate	[95% Confident Interval]	Effect model
**A vs. G**	Moore 2009	1.106	1.040-1.176	Fix
	Al Khaldi 2011	1.090	1.030-1.153	
	Dhillon2011	1.076	1.010-1.146	
	Gu 2014	1.127	1.059-1.200	
	Zhang 2015	1.066	1.001-1.135	
	Park2015	1.084	1.022-1.150	
	Hsueh 2018	1.071	1.011-1.135	
	Combined	1.088	1.029-1.151	
**AA vs. GG**	Moore 2009	1.241	1.092-1.411	Fix
	Al Khaldi 2011	1.200	1.069-1.347	
	Dhillon2011	1.159	1.021-1.315	
	Gu 2014	1.297	1.140-1.476	
	Zhang 2015	1.151	1.010-1.313	
	Park2015	1.191	1.055-1.345	
	Hsueh 2018	1.165	1.034-1.314	
	Combined	1.199	1.069-1.345	
**GA vs. GG**	Moore 2009	1.075	0.974-1.187	Fix
	Al Khaldi 2011	1.089	0.994-1.193	
	Dhillon2011	1.108	0.994-1.234	
	Gu 2014	**1.130**	**1.022-1.250**	
	Zhang 2015	1.078	0.974-1.193	
	Park2015	1.080	0.982-1.188	
	Hsueh 2018	1.063	0.966-1.168	
	Combined	1.088	0.993-1.191	
**AA+GA vs. GG**	Moore 2009	1.119	1.020-1.228	Fix
	Al Khaldi 2011	1.120	1.027-1.221	
	Dhillon2011	1.124	1.016-1.245	
	Gu 2014	1.172	1.066-1.290	
	Zhang 2015	1.098	0.998-1.209	
	Park2015	1.111	1.016-1.215	
	Hsueh 2018	**1.091**	**0.997-1.193**	
	Combined	1.118	1.026-1.219	
**AA vs. GA+GG**	Moore 2009	1.174	1.052-1.311	Fix
	Al Khaldi 2011	1.117	1.014-1.231	
	Dhillon2011	**1.080**	**0.973-1.198**	
	Gu 2014	1.169	1.048-1.303	
	Zhang 2015	**1.073**	**0.960-1.199**	
	Park2015	1.114	1.005-1.234	
	Hsueh 2018	**1.100**	**0.995-1.216**	
	Combined	1.117	1.013-1.230	

Table S3:False-positive report probability values for associations between the rs182052 polymorphism and cancer risk

Genotype	Crude OR (95% CI).	P∗	Statistical power†	Prior probability				
				.25	.1	.01	.001	.0001
Allele (A vs. G)	1.09(1.03-1.15)	0.002	1.000	0.005	0.014	0.138	0.618	0.942
Homozygous (AA vs. GG)	1.20(1.07-1.34)	0.001	1.000	0.004	0.011	0.106	0.546	0.923
Dominant (AA+GAvs. GG)	1.12(1.03-1.22)	0.009	1.000	0.027	0.078	0.482	0.904	0.989
Recessive (AA vs. GA+GG)	1.12(1.01-1.23)	0.018	1.000	0.051	0.138	0.637	0.947	0.994

OR: odds ratio,CI: confidence interval.

∗:Achi-square test was used to evaluate the distributions of genotype frequency.

†: Statistical power was calculated by use of the number of observations in the subgroup and P values in this table.

Table S4: MAFs of rs182052polymorphism in the populations from the1000 GenomesProject Phase 3

Populations	MAF
ACB	0.354
ASW	0.279
BEB	0.424
CDX	0.382
CEU	0.409
CHB	0.471
CHS	0.452
CLM	0.394
ESN	0.419
FIN	0.490
GBR	0.385
GIH	0.369
GWD	0.336
IBS	0.350
ITU	0.348
JPT	0.495
KHV	0.354
LWK	0.369
MSL	0.324
MXL	0.469
PEL	0.459
PJL	0.323
PUR	0.303
STU	0.373
TSI	0.346
YRI	0.380

MAFs:minor allele frequencies; ACB:AfricanCarribbeansinBarbados;ASW:AmericansofAfricanAncestryinSWUSA;BEB:BengalifromBangladesh;CDX:ChineseDaiinXi-shuangbanna,China;CEU:UtahResidentswithNorthernandWesternEuropeanAncestry;CHB:HanChineseinBeijing,China;CHS:SouthernHanChinese;CLM:ColombiansfromMedellin,Colombia;ESN:EsaninNigeria;FIN:FinnishinFinland;GBR:BritishinEnglandandScotland;GIH:GujaratiIndianfromHouston,Texas;GWD:GambianinWesternDivisionintheGambia;IBS:IberianPopulationinSpain;ITU:IndianTelugufromtheUK;JPT:JapaneseinTokyo,Japan;KHV:KinhinHoChiMinhCity,Vietnam;LWK:LuhyainWebuye,Kenya;MSL:MendeinSierraLeone;MXL:MexicanAncestryinLosAngelesUSA;PEL:PeruviansfromLima,Peru;PJL:PunjabifromLahore,Pakistan;PUR:PuertoRicaninPuertoRico;STU:SriLankanTamilfromtheUK;TSI:ToscaniiinItalia;YRI:YorubainIbadan,Nigeria.
